# CBCT-Based Anatomical Assessment of Lingual Bone Availability Relevant to the Mental Foramen Bypass Concept in Posterior Atrophied Mandibles

**DOI:** 10.3390/jcm15187011

**Published:** 2026-09-10

**Authors:** Abduljaleel Samad, Omed Shihab, Fedil Yalda, Jodal Ahmed, Azhin Raza

**Affiliations:** 1 College of Dentistry, Hawler Medical University, Erbil 44001, Kurdistan Region, Iraq; omed.shahb@hmu.edu.krd (O.S.); fedil.yalda@hmu.edu.krd (F.Y.); jodal.ahmed@hmu.edu.krd (J.A.); 2 Ashti Hospital, Soran Health Directorate, Soran 44008, Kurdistan Region, Iraq; ajin.mawa@gmail.com

**Keywords:** mental foramen bypass, prosthetically driven implant placement, atrophied mandible, dental implants, cone-beam computed tomography, anterior loop, implant rehabilitation

## Abstract

**Background/Objectives:** Severe posterior mandibular atrophy presents anatomical challenges for implant rehabilitation because of reduced residual bone volume and the close relationship between the mental foramen, its anatomical variations, and the surrounding alveolar bone. Among these variations, the anterior loop may further influence the available lingual bone adjacent to the mental foramen. Despite the clinical importance of this region, quantitative three-dimensional anatomical information regarding lingual bone availability remains limited. Therefore, the aim of this retrospective cone-beam computed tomography (CBCT)-based study was to quantitatively evaluate the availability of lingual bone adjacent to the mental foramen in posterior atrophied mandibles and to assess the influence of the anterior loop on these anatomical dimensions, thereby providing quantitative anatomical information about this region. **Methods:** In a retrospective CBCT-based anatomical study, 110 CBCT scans of adult patients with atrophied mandibles were analyzed using standardized cross-sectional views perpendicular to the dental arch; linear measurements were obtained from the lingual cortical plate to the mesial (Point A), middle (Point B), and distal (Point C) borders of the mental foramen. The presence of the anterior loop was recorded. Descriptive statistics and independent-samples *t*-tests were performed to compare measurements between loop and no-loop groups (α = 0.05). **Results:** The mean measurements of the lingual bone were 7.22 ± 1.65 mm at Point A, 6.18 ± 1.55 mm at Point B, and 4.82 ± 1.38 mm at Point C, showing a gradual decrease from mesial to distal. An anterior loop was found in 26.4% of the cases. Significantly thinner dimensions of the lingual bone were observed with an anterior loop at all three measurement points (*p* < 0.01). **Conclusions:** The present CBCT-based anatomical study measured the amount of lingual bone available adjacent to the mental foramen in posterior atrophied mandibles and observed a significant correlation between the presence of an anterior loop and reduced lingual bone dimensions. These findings add to the knowledge of the anatomy of this area and could serve as the basis for future clinical studies to investigate the potential for a mental foramen bypass concept.

## 1. Introduction

Rehabilitation of the atrophied posterior mandible represents a continuous challenge in implant dentistry due to progressive alveolar bone resorption and its close anatomic relationship with the inferior alveolar nerve [1]. The presence of the mental foramen and its possible anatomical variations in the premolar region may limit the available bone height and width and increase neurosensory complications due to improper assessment and planning [2].

The mental foramen region represents one of the most anatomically complex areas of the posterior mandible because of the close anatomical relationship among the mental foramen, the inferior alveolar canal, the anterior loop, and the surrounding alveolar bone [2].

The morphology, position and anatomical features of these structures have been found to greatly vary from one person to another, especially in terms of the mental foramen and anterior loop, which may affect the quantity and distribution of the lingual bone adjacent to the mental foramen [3]. Consequently, detailed three-dimensional anatomical characterization of this region is essential for improving the understanding of posterior mandibular morphology and for establishing reliable anatomical reference data that may serve as a foundation for future anatomical and clinical investigations [4].

The most common location of the mental foramen is close to the apex of the second premolar, but there may be variations in different populations and between individuals [5,6]. Other variations also reported include accessory mental foramina and the anterior loop of the mandibular canal. An anterior loop represents an anterior extension of the inferior alveolar nerve beyond the mental foramen before looping back to exit, and prevalence and length are very variable between populations [7,8,9]. If this anatomical variation is not identified, altered sensation, paresthesia, or permanent nerve damage could be a consequence of implant placement. Despite the recognized anatomical variability of this region, the dimensions of the lingual bone adjacent to the mental foramen have received relatively little attention in the literature.

To deal with limited bone height and width in the posterior mandible, several surgical approaches have been suggested, including short implants, tilted implants, vertical ridge augmentation, distraction osteogenesis, and inferior alveolar nerve lateralization or transposition. Although these techniques are regarded as effective, they are most of the time associated with increased surgical complexity, higher morbidity, prolonged treatment time, and a higher risk of neurosensory disturbances [10].

Cone-beam computed tomography has become the imaging modality of choice for three-dimensional anatomical assessment of the jaws because it provides reliable linear measurements of related anatomical structures and details about cortical and trabecular bone morphology with relatively low radiation exposure [11]. It allows for precise evaluation of the buccolingual position of the mandibular canal, the presence of an anterior loop, and the available bone dimensions surrounding the mental foramen.

To the authors’ knowledge, this is the first cone-beam computed tomography-based study to quantitatively evaluate lingual bone availability relevant to mental foramen bypass concept in atrophied mandibles while correlating measurements with the presence of an anterior loop.

The aim of the present retrospective CBCT study was to evaluate the availability of lingual bone around the mental foramen in posterior atrophied mandibles and to assess the effect of the anterior loop on these anatomical dimensions. The purpose of the present study was to explore the anatomical basis of the mental foramen bypass concept and to provide the anatomical information for further investigation of the mental foramen bypass concept. The present study was limited to anatomical evaluation by CBCT and was not intended to evaluate clinical applicability or treatment effectiveness.

## 2. Materials and Methods

### 2.1. Study Design and Ethical Approval

This retrospective CBCT-based anatomical study was conducted at the College of Dentistry, Hawler Medical University, Erbil, Iraq. The study protocol was reviewed and approved by the Scientific Research Ethical Committee of the the College of Dentistry, Hawler Medical University, Erbil, Iraq (Reference No. HMUD/2526009; approval date: 5 October 2025).

All cone-beam computed tomography data were anonymized prior to analysis, and the study was conducted according to international ethical guidelines.

### 2.2. Sample Selection

A total of 110 CBCT scans of adult patients involving the posterior mandible were included in the study. Inclusion criteria were: age ≥18 years, presence of an atrophied posterior mandible and clear visualization of the mental foramen region.

In this retrospective CBCT study, posterior mandibular atrophy was operationally defined as ≤4 mm residual crestal width at the planned implant site on CBCT. This threshold was chosen because standard diameter implants usually require adequate surrounding bone, with approximately 5 mm of ridge width considered acceptable for implants measuring 3.5–4.2 mm in diameter, while contemporary implant planning recommends maintaining approximately 1.0–1.5 mm of bone around the implant [12]. Therefore, a residual ridge width ≤4 mm was deemed an appropriate operational threshold for identification of the clinically challenging posterior mandibular anatomy and for assessment of the available lingual bone adjacent to the mental foramen.

Exclusion criteria included the presence of pathological lesions in the premolar region, previous mandibular surgery or trauma, and poor image quality or artifacts affecting measurements.

### 2.3. Cone-Beam Computed Tomography Evaluation and Measurements

Cone-beam computed tomography images were analyzed using standardized cross-sectional views perpendicular to the dental arch. Measurements were obtained from the lingual cortical plate to the mental foramen at three reference points: Point A: Most mesial border of the mental foramen. Point B: Midpoint of the mental foramen. Point C: Most distal border of the mental foramen.

**Figure 1:**
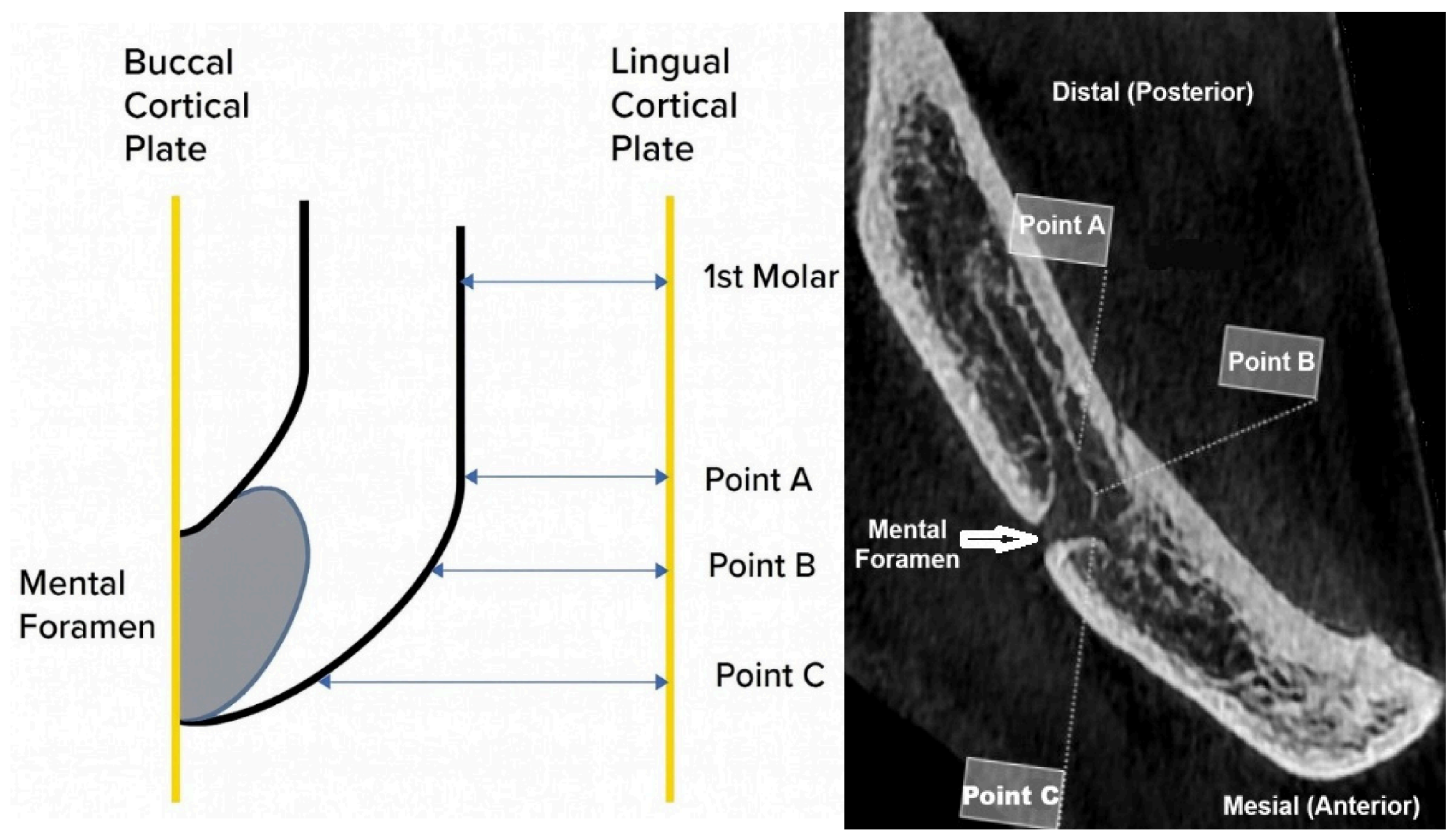
Measurements on cone-beam computed tomography were obtained from the lingual cortical plate to the mental foramen at three reference points: Point A: Most mesial border of the mental foramen. Point B: Midpoint of the mental foramen. Point C: Most distal border of the mental foramen.

Both right and left sides were measured when available. The presence and laterality of the anterior loop (absent, right, left, bilateral) were recorded (Figure 1).

### 2.4. Cone-Beam Computed Tomography Scanner Details

All CBCT scans were taken using a NewTom GIANO cone-beam computed tomography (QR S.r.l., Verona, Italy). Focused field-of-view scans provided high-resolution images of the mandibular premolar and molar regions; thus, the mental foramen, anterior loop, and adjacent cortical bone could be measured with high accuracy.

The scans were acquired using Focused field-of-view (FOV) of 5 × 11 cm with a voxel size of 0.125 mm in High Resolution mode with exposure parameters (90 kV, 8.0 mA and 9.0 s). Image reconstruction and analysis were done using the manufacturer’s proprietary NNT software (version 15.3; NewTom, QR S.r.l., Verona, Italy). Multiplanar reconstructions were generated in axial, sagittal, and cross-sectional views, 1 mm thick and 1 mm apart, oriented perpendicular to the mandibular arch.

### 2.5. Inter- and Intra-Observer Reliability

All measurements were performed independently by two calibrated examiners (an oral and maxillofacial surgeon and an oral and maxillofacial radiologist), both experienced in CBCT interpretation and implant planning.

Prior to the main analysis, the observers underwent a calibration session using 15 randomly selected CBCT scans that were not included in the final study sample. Measurement protocols, anatomical landmarks (Points A, B, and C), and slice orientation were standardized and discussed until consensus was achieved. Measurements were repeated until intra-observer variation was minimized.

To assess intra-observer reliability, each examiner repeated all measurements on the same 15 cone-beam computed tomography scans after a two-week interval, under identical viewing conditions and without access to the initial results. Intra-class correlation coefficient analysis demonstrated excellent intra-observer agreement (ICC = 0.91–0.94).

Inter-observer agreement was determined by comparing the measurements taken independently by the two observers for the same group of CBCT scans, demonstrating excellent inter-observer agreement (ICC = 0.88–0.92).

### 2.6. Statistical Analysis

Data analysis was done using the IBM SPSS Statistics for Windows, Version 26.0 (IBM Corp., Armonk, NY, USA). Descriptive statistics including the mean, standard deviation, minimum, maximum values were calculated. Independent *t*-test analysis was conducted to evaluate the measurement between the loop and no-loop group. Statistical significance was set at *p* < 0.05.

## 3. Results

### 3.1. Study Sample Characteristics

A total of 110 CBCT scans were included in the analysis. The study population consisted of 48 males (43.6%) and 62 females (56.4%) (Table 1).

The age distribution showed that the majority of patients were in the 20–29 years age group (36.4%), followed by 40–49 years (16.4%), 30–39 years (15.5%), and 50–59 years (14.5%). Smaller proportions were observed in patients aged <20 years and ≥70 years (each 4.5%) (Table 2).

The anterior loop of the mental nerve was absent in 81 cases (73.6%). An anterior loop was identified in 29 cases (26.4%), including 5 cases on the right side (4.5%), 6 cases on the left side (5.5%), and 18 cases with bilateral presentation (16.4%) (Table 3).

### 3.2. Descriptive Statistics of Lingual Bone Measurements

Lingual bone measurements showed a reduction from the mesial to the distal aspect of the mental foramen.

- On the right side, mean values were:

7.22 ± 1.66 mm at Point A (range 3.70–11.50 mm),

6.18 ± 1.55 mm at Point B (range 2.20–9.70 mm),

4.88 ± 1.38 mm at Point C (range 1.50–9.00 mm).

- On the left side, mean values were:

7.22 ± 1.58 mm at Point A (range 3.70–10.50 mm),

6.10 ± 1.51 mm at Point B (range 1.20–10.00 mm),

4.83 ± 1.43 mm at Point C (range 1.00–8.80 mm).

Complete descriptive statistics for all measurement points and sides are presented in (Table 4).

Comparative analysis revealed statistically significant differences in lingual bone dimensions between cases with and without an anterior loop at all measured points (Table 5).

At Point A, the mean measurement was 6.24 ± 1.40 mm in the loop group compared to 7.49 ± 1.57 mm in the no-loop group, with a mean difference of −1.25 mm (*p* < 0.001).

At Point B, mean values were 5.46 ± 1.26 mm in the loop group and 6.32 ± 1.55 mm in the no-loop group, with a mean difference of −0.86 mm (*p* = 0.001).

At Point C, the loop group demonstrated a mean of 4.07 ± 1.16 mm, compared to 5.07 ± 1.39 mm in the no-loop group, resulting in a mean difference of −0.99 mm (*p* < 0.001).

Overall, the presence of an anterior loop was consistently associated with significantly smaller lingual bone dimensions at all three reference points.

## 4. Discussion

The present research study provides quantitative CBCT-based anatomical assessment regarding the availability of lingual bone adjacent to the mental foramen in posterior atrophied mandibles. The mean measurements at Points A and B indicate that the lingual bone dimensions were greater at the mesial and middle aspects of the mental foramen than at the distal aspect. These findings add to the anatomical description of the region of the posterior mental foramen and to the quantitative data on lingual bone morphology. These findings should be viewed as anatomic observations, not evidence of clinical efficacy.

Previous reports have stated that buccolingual bone width in the posterior mandible is frequently underestimated by two-dimensional diagnostic imaging. Three-dimensional analysis has shown that the lingual cortical plate can offer a stable anchorage position when the buccal vertical bone height is insufficient for implant placement [13,14,15]. These findings are in line with previous reports highlighting the usefulness of CBCT for the 3D anatomical evaluation of the posterior mandible in the proximity of vital anatomical structures.

The decrease in the lingual bone dimensions seen in association with the existence of an anterior loop highlights the anatomical importance of this anatomical variation. There are reports by various authors indicating that the anterior loop represents the most serious risk factor for neurosensory complications with implant placement into the premolar region [16,17].

One of the most clinically relevant findings in the present investigation was the statistically significant reduction in lingual bone dimensions in cases presenting with an anterior loop, consistently seen at all measured points (Table 5). The mean lingual bone thickness at Point A, located at the mesial aspect of the mental foramen, was 6.24 ± 1.40 mm for the loop group, versus 7.49 ± 1.57 mm for the no-loop group, indicating a mean difference of −1.25 mm (significant, *p* < 0.001).

At the midpoint of the mental foramen, at Point B, cases with an anterior loop had a mean lingual bone dimension of 5.46 ± 1.26 mm, which is significantly low compared with 6.32 ± 1.55 mm recorded in cases without a loop, with a mean difference of −0.86 mm (*p* = 0.001). At the distal aspect (Point C), where bone availability is already limited, the mean values were 4.07 ± 1.16 mm in the loop group versus 5.07 ± 1.39 mm in the no-loop group, which means a further reduction by the presence of an anterior loop (mean difference −0.99 mm, *p* < 0.001).

These findings indicate that the anterior loop is more than just a neuroanatomical variation. Previous CBCT reports have indicated that an association exists between the presence of an anterior loop with altered canal trajectory and reduced surrounding bone envelope, particularly in the premolar region [16,17,18,19]. The present study quantitatively confirms this relationship and extends it by demonstrating the impact of the anterior loop across the entire mesiodistal extent of the mental foramen. These findings indicate an association between the presence of an anterior loop and reduced lingual bone dimensions in the measured region.

These results further strengthen the importance of routine CBCT evaluation, especially when detailed assessment of anatomical variations is needed. Reliance upon two-dimensional imaging may result in failure to detect not only the anterior loop itself but also its effects on the morphology of the surrounding bone and hence may increase the potential for iatrogenic complications [15,20].

Interestingly, a considerable proportion of the subjects in the present study were quite young despite the presence of posterior mandibular atrophy. These findings suggest that residual ridge resorption is affected by several factors apart from chronological age, such as the duration of edentulism, periodontal disease, traumatic tooth loss, occlusal loading, and individual variations in bone remodeling [21,22]. Thus, the degree of posterior mandibular atrophy should not be determined by age alone, highlighting the importance of a customized 3D-CBCT evaluation during implant treatment planning.

The present study has several limitations that should be considered when interpreting the findings. It was mainly a retrospective CBCT-based anatomical study and thus provides quantitative anatomical information only. The study was not intended to evaluate the clinical efficacy, safety, predictability, or treatment results of the proposed mental foramen bypass concept. Second, the study population was derived from a single center, which may limit the generalizability of the findings to other populations with different anatomical characteristics. Third, a working definition of posterior mandibular atrophy was chosen for this investigation: posterior mandibular atrophy was operationally defined on CBCT as residual crestal width ≤4 mm. Because no universally accepted quantitative definition of posterior mandibular atrophy based solely on ridge width currently exists, this criterion should be interpreted within the context of the present study. Furthermore, the potential influence of patient-related variables (i.e., age, sex, degree of edentulism and side (right/left)) on the measured anatomical dimensions was not specifically explored. Since the study was designed to characterize lingual bone availability and its relationship with the anterior loop, it was not statistically powered to evaluate these variables as independent predictors. Future multicenter prospective clinical studies involving larger and more diverse populations are warranted to validate these anatomical observations and to determine their potential clinical relevance.

The anatomical observations reported in the present study identify a previously underexplored region of lingual bone adjacent to the mental foramen in posterior atrophied mandibles. These findings may provide a rationale for future prospective clinical studies designed to evaluate whether this anatomical pathway has potential clinical relevance. Until such evidence becomes available, the present results should be interpreted solely as anatomical observations derived from CBCT analysis and should not be considered recommendations for patient selection, surgical planning, or treatment decision-making.

## 5. Conclusions

The present retrospective CBCT-based anatomical study provides quantitative information regarding the availability of lingual bone adjacent to the mental foramen in posterior atrophied mandibles and identifies a significant association between the presence of an anterior loop and reduced lingual bone dimensions. These findings contribute to the anatomical understanding of the mental foramen region and provide an anatomical basis for future prospective clinical studies investigating the potential clinical relevance of the mental foramen bypass concept.

## Figures and Tables

**Table 1:** Demographic distribution of the study sample.

Category	Frequency (n)	Percentage (%)
Male	48	43.6
Female	62	56.4
Total	110	100.0

**Table 2:** Age distribution of the study sample.

Age Groups	Frequency (n)	Percentage (%)
<20 years	5	4.5
20–29 years	40	36.4
30–39 years	17	15.5
40–49 years	18	16.4
50–59 years	16	14.5
60–69 years	9	8.2
≥70 years	5	4.5
Total	110	100.0

**Table 3:** Distribution of anterior loop of the mental nerve.

Anterior Loop Status	Frequency (n)	Percentage (%)
Absent	81	73.6
Right side	5	4.5
Left side	6	5.5
Bilateral	18	16.4

**Table 4:** Lingual bone measurements according to reference point and side (mm).

Measurement Point	Side	Minimum	Maximum	Mean	SD
Point A	Right	3.70	11.50	7.22	1.66
Point B	Right	2.20	9.70	6.18	1.55
Point C	Right	1.50	9.00	4.88	1.38
Point A	Left	3.70	10.50	7.22	1.58
Point B	Left	1.20	10.00	6.10	1.51
Point C	Left	1.00	8.80	4.83	1.43

**Table 5:** Comparison of lingual bone measurements between loop and no-loop groups.

Point	Group	Mean (mm)	SD	Mean Difference	*p*-Value
A	With loop	6.24	1.40	−1.25	<0.001
A	No-loop	7.49	1.57		
B	With loop	5.46	1.26	−0.86	0.001
B	No-loop	6.32	1.55		
C	With loop	4.07	1.16	−0.99	<0.001
C	No-loop	5.07	1.39		

## Data Availability

The data that supports the findings of this research are accessible from the corresponding author upon reasonable request and subject to institutional ethics and privacy restrictions.

## Abbreviation

The following abbreviation is used in this manuscript:

CBCT	Cone-Beam Computed Tomography
